# Vitamin E Supplementation Increases the Attractiveness of Males' Scent for Female European Green Lizards

**DOI:** 10.1371/journal.pone.0019410

**Published:** 2011-04-28

**Authors:** Renáta Kopena, José Martín, Pilar López, Gábor Herczeg

**Affiliations:** 1 Behavioural Ecology Group, Department of Systematic Zoology and Ecology, Eötvös Loránd University, Budapest, Hungary; 2 Departamento de Ecología Evolutiva, Museo Nacional de Ciencias Naturales, Consejo Superior de Investigaciones Científicas, Madrid, Spain; 3 Ecological Genetics Research Unit, Department of Biosciences, University of Helsinki, Helsinki, Finland; UC Santa Barbara, United States of America

## Abstract

**Background:**

In spite that chemoreception is important in sexual selection for many animals, such as reptiles, the mechanisms that confer reliability to chemical signals are relatively unknown. European green lizards (*Lacerta viridis*) have substantial amounts of α-tocopherol ( = vitamin E) in their femoral secretions. Because vitamin E is metabolically important and can only be attained from the diet, its secretion is assumed to be costly. However, its role in intraspecific communication is unknown.

**Methodology/Principal Findings:**

Here, we experimentally show that male European green lizards that received a dietary supplement of vitamin E increased proportions of vitamin E in their femoral secretions. Furthermore, our experiments revealed that females preferred to use areas scent marked by males with experimentally increased vitamin E levels in their secretions. Finally, female preferences were stronger when vitamin E differences between a pair of males' secretions were larger.

**Conclusions/Significance:**

Our results demonstrate that female green lizards are able to discriminate between males based on the vitamin E content of the males' femoral secretions. We suggest that the possible cost of allocating vitamin E to secretions, which might be dependent on male quality, may be a mechanism that confers reliability to scent marks of green lizards and allows their evolution as sexual signals.

## Introduction

Theoretical models predict that sexual signals can only be evolutionarily stable if they are honest, i.e., condition dependent and costly to the signaler, and if the signal's cost is correlated with the signaler's quality (e.g. [1]–[2], but see [3]–[5] for more complex scenarios). Production of chemical signals may be energetically costly, especially if chemicals cannot be produced by the animals themselves, but have to be acquired from food [6]–[7]. Such chemical signals can be used as ‘honest signals’ (*sensu* [8]) or ‘revealing indicators’ (*sensu* [9]–[10]), which provide reliable information, as they would accurately reflect the signaler's ability to exploit resources [11]. Chemical signals of territorial animals can have further ecological costs [6]: (i) time and energy investments to depositing and maintaining the signals and (ii) the increased of risk of predation and parasite infection during deposition and maintenance.

Irrespective of the costs, chemical signals play an important role in intraspecific communication and sexual selection of a wide array of taxa and contexts (e.g. [6], [12]–[15]). In lizards, pheromonal detection is often based on femoral gland secretions ([16]–[18] reviewed in [13]), which may be used in sexual selection [7], [18]–[20]. Behavioral experiments suggested that femoral gland secretions might transmit information about a male's quality, and thus females may use this information to choose their mates [7], [19], [21]–[22]. However, the chemical composition of gland secretions is rarely studied [23], and the mechanisms that could confer reliability to chemicals as sexual signals remain poorly understood.

Different species of so-called ‘green lizards’ have substantial amounts of α-tocopherol ( = vitamin E) in their femoral secretions (*Lacerta schreiberi* [24]; *L. viridis* [25]; *L. lepida* [26]). Vitamin E consists of a group of isoprenoid compounds of plant origin that are the main lipophilic antioxidants and radical scavengers involved in membrane defense, and that also have immunostimulatory activity [27]–[29]. Secretion of vitamin E is assumed to be costly for lizards: α-tocopherol is typically produced by microorganisms and plants, and thus it should be of dietary origin [30]. The physiological relevance of vitamin E and the severe pathological consequences of its deficiency, such as neurological disorders or lung diseases, together with its dietary origin impose a major challenge for sustaining an adequate supply of this vitamin to different tissues [31]. Because vitamin E is important metabolically, it should only be allocated to femoral secretions when it is in abundant supply.

We tested the hypothesis that high levels of vitamin E in femoral secretions of male European green lizards (*L. viridis*) might be a sexual trait preferred by females. We supplemented experimental males with dietary vitamin E, and examined (i) whether the treatment changed the characteristics of their femoral secretions, (ii) whether females were more attracted to areas scent marked by supplemented than non-supplemented control males, and (iii) if the strength of females' preferences were related to the magnitude of differences in vitamin E levels between males offered. We predicted that vitamin E supplementation will enrich the vitamin E content of male European green lizards' femoral secretions, that females will prefer males with more vitamin E in their secretions, and that the strength of female preference is positively correlated with the vitamin E level difference between the offered male secretions.

## Methods

### Ethics statement

Experiments were performed according to the guidelines of the Hungarian Act of Animal Care and Experimentation (1998, XXVIII, section 243/1998), which conforms to the regulation of animal experiments by the European Union. The experiment was done under the license of the Middle-Danube-Valley Inspectorate for Environmental Protection, Nature Conservation and Water Management (No. 21765/2007). All animals were returned healthy to their capture sites at the end of the experiment.

### Study animals

We used noosing to capture 36 adult European green lizards (16 females and 20 males) at the beginning of April 2007, before the start of their mating season, near Tápiószentmárton (Pest County, Hungary; 47°20′N, 19°47′E). The habitat is sand ‘puszta’ with disturbed grassland, honey locust (*Gleditsia triacanthos*) scrub, and black pine (*Pinus nigra*) forest patches. Only adult lizards with intact or fully regenerated tails were considered.

Lizards were transported to the Zoological Institute of the Szent István University, and housed individually in indoor 70×40×50 cm (length, width, and height, respectively) plastic terraria. Lizards were fed mealworms and crickets and water was provided *ad libitum*. Terraria of males and females were in different rooms to avoid any contact between them before trials. The room temperature was roughly constant (28°C) and the photoperiod was held natural (approx. 14L∶10D).

### Vitamin E supplementation

We made the experiments in May 2007. We paired males (10 pairs) based on their snout-to-vent length (maximum difference was 0.6 mm). One male of each pair was assigned randomly to the supplement treatment, and the other to the control treatment. Supplemented males (S-males) received daily, during 21 days, 0.04 ml of vitamin E supplement (synthetic (±)-α-tocopherol; purchased from Sigma-Aldrich Chemicals Co.), which contained 97% of synthetic vitamin E (approx. 1014 IU ml^−1^) and 3% soybean oil (with approx. 0.32 IU ml^−1^ of natural vitamin E, i.e. D-α-tocopherol). Thus, we provided S-males with approximately 40.5 IU of vitamin E per dose. While this dose is above the daily minimal physiological necessity of vitamin E for reptiles of this size, it is still well below the tolerable upper intake leves [30], [32]–[33]. To ensure that all lizards swallowed the entire dose, we gently handled lizards and used sterile plastic syringes with a canula to deliver slowly the solution into their mouth. Control males (C-males) were handled in the same way as S-males, but we administered them 0.04 ml of distilled water instead of the vitamin supplement to avoid any unwanted vitamin E intake. Therefore, the soybean oil in the diet also varied between treatments. However, as all experimental animals were fed *ad libitum*, we believe that the very small amount of supplemented oil *per se* could not have an effect on the quality or quantity of the males' secretion.

### Chemical analyses of male femoral secretions

At the end of the experiments, we collected femoral secretion of males directly into glass vials with Teflon-lined stoppers that were stored at −20°C. Samples were analyzed by gas chromatography-mass spectrometry (ThermoQuest Trace 2000) equipped with a Supelco-Equity-5 column temperature programmed (50–280°C at 5°C/min and 280°C for 30 min). Compounds were identified by comparison of mass spectra in the NIST library, and later confirmed with authentic standards (see [25] for details of analyses and chemicals in secretions). The relative amount of each component was determined as the percent of the total ion current (TIC) area transformed following Aitchison's formula [34]: [*Z_ij_*_,_ = *ln (Y_ij_/g(Y_j_)*], where *Z_ij_* is the standardized peak area *i* for individual *j*, *Y_ij_* is the peak area *i* for individual *j*, and *g*(*Y_j_*) is the geometric mean of all peaks for individual *j* (for similar analyses see [35]). Then, we calculated the relative proportions of the different types of chemicals in secretions (alcohols, fatty acids, lactones, steroids, squalene, and α-tocopherol; see [25]). To test for differences in relative proportions of chemicals in secretions of S- and C- males, we ran a multivariate General Lineal Model (GLM) with transformed areas of alcohols, fatty acids, lactones, steroids, squalene, and α-tocopherol ( = vitamin E). Then, because we found a significant result in the multivariate analyses (see results), we could perform protected GLMs separately on each chemical or groups of chemicals, to test which one changed more with the diet supplementation and explained the significant difference found in the previous multivariate GLM model [36]. Following the significant findings (see Results), we also ran a repeated measures GLM using α-tocopherol ( = vitamin E) proportions as the dependent variable, and male treatment (S *vs.* C) within the size-matched pairs (see above) used in the preference tests as a repeated measures factor.

### Female preference tests

During the last week of the experimental supplementation, we placed eight absorbent paper stripes (6×40 cm) on the floor of males' terraria, and left them there to obtain the scents from femoral secretions of each male. Food was not deposited on the papers to avoid odor contamination. During this week, S-males were still receiving the supplement of dietary vitamin E and C-males the water.

Female preference tests were performed at the end of this exposition period. Females' plastic terraria (70×40×50 cm, length, width, height, respectively) had three water dishes and three cardboard shelters that also acted as basking places, two placed symmetrically at each end of the cage and one in the centre. We tested all females with five different male pairs in five consecutive days. We chose male pairs and females randomly, but in a way that every male pair was tested eight times with different females and every female met five different pairs of males. At the beginning of each experiment (0800 h GTM), when females were still inactive, we took a paper strip from each of the two males from a given pair (one C-male and one S-male matched in size; see above) and fixed their strips on alternate ends of the female's terraria (side was chosen randomly). Paper strips were handled with fresh gloves to avoid contamination with human odor.

Females were monitored every 10 min from a concealed view point and their locations (determined by the head position) in the terraria were recorded from 0900 to 1400 h GTM. We divided terraria into three equally-sized parts: two terminal parts where scent-marked papers were located, and a central ‘neutral’ part. We excluded trials where females (i) were observed only in one area or (ii) were located in the area of each male's scent less times than on the area of the other male and the neutral area together, to ensure that females were exposed to both males' tiles and were aware of both male's stimuli, and to avoid trials were females might be stressed by the setup and did not respond.

We analyzed the outcome of the female preference tests with Generalized Linear Mixed Models (GLMMs) with binomial error and logit link. First, we ran a GLMM with the proportion a certain female was observed at a certain male's side (N observations = 30, see above) as dependent variable, treatment (supplemented *vs.* control) and the identity of both the females and the male pairs as fixed factors, and trial (the experiment including a certain male pair and a certain female) as random factor. Second, we ran another GLMM to test whether the strength of female preference was related to the vitamin E difference within a given male pair. Here, we used the mean of the proportions of female observations at a certain male's area (every male pair was assessed by several females) as dependent variable, the vitamin E concentration in the given male's secretion as a continuous fixed effect, and male pair as random factor. We note that we also ran two simple repeated measures GLMs built similarly to the two GLMMs above, but with the arcsine-squareroot transformed proportion of female observations as dependent variable, and male treatment (S *vs.* C) within the size-matched pairs as repeated measures factor. These models revealed qualitatively similar patterns to the GLMMs (data not shown).

## Results

### Chemicals in femoral secretions

After the experimental supplementation, chemicals found in femoral secretions of both groups of males were qualitatively similar (i.e., the same chemical compounds were found in secretions of both C- and S-males), but S-males had secretions that differed in relative proportion of chemicals from those of C-males (multivariate GLM, Wilks'λ = 0.18, *F*_6,13_ = 9.71, *P*<0.001). However, these differences were only explained because S-males had significantly higher proportions of α-tocopherol ( = vitamin E) (GLM, *F*_1,18_ = 15.48, *P*<0.001; mean ± SE transformed TIC areas, S-males = 5.67±0.12; C-males = 4.86±0.12). In contrast, there were no significant differences between S- and C-males in proportions of alcohols (*F*_1,18_ = 0.01, *P* = 0.93), fatty acids (*F*_1,18_ = 0.81, *P* = 0.38), lactones (*F*_1,18_ = 1.06, *P* = 0.32), steroids (*F*_1,18_ = 2.83, *P* = 0.11) or squalene (*F*_1,18_ = 1.68, *P* = 0.21). Therefore, the experimental supplementation only affected to contents of vitamin E in secretions, which were otherwise similar between treatments in their chemical composition and proportion of chemicals.

In addition, if we compared differences in vitamin E content between the S- and C-male within each pair, we also found a significant difference (repeated measures GLM, *F*_1,9_ = 40.84, *P*<0.001). However, the variation in vitamin E differences within male pairs was considerable (range of differences in TIC transformed areas = 0.17–1.24), probably due to individual differences in initial proportions of vitamin E in the males of a pair.

### Female preference tests

**Figure 1:**
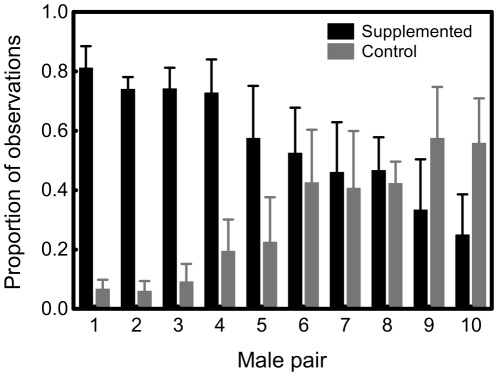
Female preference for male scent. The proportion unmated female lizards (see text for details) were observed at the areas containing chemical cues from size-matched vitamin E supplemented *vs.* control male European green lizards. Means for every male pair (± SE) are shown.

**Figure 2:**
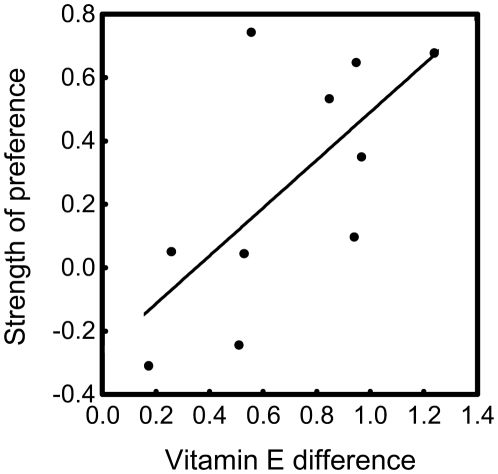
Strength of female preference for male scent. Female ‘strength of preference’ was calculated for every male pair as the difference in the mean proportion unmated female lizards (see text for details) were observed at the areas containing chemical cues from size-matched vitamin E supplemented *vs.* control male European green lizards. Vitamin E difference is the difference in relative vitamin E content of the femoral secretions of size-matched vitamin E supplemented *vs.* control males within a male pair.

Our first GLMM revealed that the treatment effect was male pair dependent (treatment: F_1,36_<0.01, P>0.99, male pair: F_9,36_ = 0.11, P>0.99, female: F_14,36_ = 0.08, P>0.99, treatment×male pair: F_9,36_ = 3.60, P = 0.003, treatment×female: F_14,36_ = 1.51, P = 0.16). Removal of the nonsignificant effects including the factor ‘female’ resulted in a significant treatment effect while retaining the treatment×male pair interaction (treatment: F_1,50_ = 11.77, P = 0.0012, male pair: F_9,50_ = 0.07, P>0.99, treatment×male pair: F_9,50_ = 2.29, P = 0.031). Females were observed more times in the areas of vitamin E supplemented males than in the areas of control males; however, the effect varied among male pairs (Figure 1). Further, our second GLMM revealed that the attractiveness of a certain male's area depended on the vitamin E concentration difference of the males' femoral secretion within a pair (F_1,18_ = 9.17, P = 0.007): larger difference resulted in stronger preference (Figure 2).

## Discussion

Our results supported our predictions: (i) dietary vitamin E supplementation increased the vitamin E proportions in the femoral secretions of male European green lizards, (ii) female lizards were spatially associated to the scent of vitamin E supplemented males, and (iii) the strength of female preference was positively correlated to the vitamin E level differences between the offered scents of supplemented *vs.* control males.

The effect of dietary vitamin E supplementation on the femoral secretions' composition suggests that lizards with higher quality diets are able to divert more vitamin E from metabolism into their chemical signals. This, together with the fact that α-tocopherol is of dietary origin suggests that allocation of vitamin E to secretions might be costly and dependent of male quality. The nutritional condition of males also affects the attractiveness of their pheromones to females in some insects [37]–[38], and vitamin D supplementation increased the quality of femoral secretions in Iberian rock lizards (*L. monticola* [7]).

Female European green lizards showed clear preference to use areas scent marked by males with more vitamin E in their secretions, especially when the vitamin E divergences between available scents were large. The reported female preference may be explained by at least four ways. First, females might directly use this male trait (i.e., higher levels of vitamin E) as a reliable advertisement of male quality, because higher levels of vitamin E in secretions may, for example, be correlated with the quality of the immune systems of males, as it occurs in another green lizard species (*L. lepida*; [26]). Second, females might use this trait indirectly to estimate the quality of the males' territory (i.e., quality of available food). Hence, females may use the performance of males as public information for habitat assessment [39]. Third, females might not directly use vitamin E levels as signals, but simply prefer scent marks with high vitamin E levels because vitamin E is an antioxidant that would increase duration and intensity of information provided by other chemicals in secretions [40], which might include other actually signaling chemicals. Finally, females might have a sensory bias and be attracted to vitamin E because this could be a food stimulus, indicating the presence of food, independently of the male signal [41]. Distinguishing between these scenarios requires further experiments. However, it is likely that only high quality males might allow investing high amounts of vitamin E into their secretions and be able to attract females to their territories. Therefore, we suggest that the cost of allocating vitamin E to secretions, which should be differentially costly for different individual males, may be a mechanism that confers reliability to scent marks of green lizards and allows their evolution as sexual signals. Nevertheless, we cannot exclude an alternative scenario about the role of vitamin E supplementation. In theory, it might be possible that the extra dietary vitamin E increased not only the relative amount of vitamin E in the secretion, but the total quantity of secretion itself too, and that female European green lizards might be associated with larger amounts of secretion from experimental males rather than with secretions with relatively higher vitamin E content. However, the amount of femoral secretion produced is under direct androgenic control in lizards, such that androgen treated males have higher secretion rates independently of the diet [reviewed in 13], [ 20]. Moreover, chemosensory responses of female Iberian rock lizards to scent of males or mixes of compounds seem related to the composition of the chemical stimulus, rather than to the total quantity of the chemical stimulus (19, unpublished data ), making the above scenario unlikely.

Sexual selection and female mate choice within it are widely studies topics in a number of taxa [42]. Surprisingly, such studies on reptiles are scarce, despite the wide array of potential signals (colorful ornaments, chemical signals, behavioral displays) that reptiles, and especially lizards exhibit (e.g. [25], [43]–[46]). Unlikely in many taxa [42], female mate choice has rarely been reported in lizards [47]–[48]. Mating seems to be under strict male control in non-territorial lizards [49] and female mate choice is unlikely to be important in such systems (but see [50]) where females copulate with multiple males and have the potential to choose only at the sperm level [47]–[48]. However, in lizards where males are territorial (like the European green lizard) females have the chance to choose their reproductive partner either according to its own quality [35], [41], [45], [51] or indirectly, according to the quality of its territory [52]. Further, considering visual *vs.* chemical signals, females can choose both in the presence and absence of males. The vitamin E content based female preference reported here suggests that female European green lizards can choose their potential reproductive partner in the absence of it, however, we do not know if vitamin E levels reflect the quality of the males, of their territories or both. A recent study reported female mate preference based on nuptial throat coloration (ultraviolet) in European green lizards [53]. Hence, it seems that females of this lizard species have multiple ways and means to assess the quality of their potential reproductive partners.

In summary, we found that the vitamin E content of the femoral gland secretion of male European green lizards depends on their food, and that females preferred to associate with male scents with high vitamin E content. We suggest that the possible – male quality dependent – cost of allocating vitamin E to secretions may be a mechanism that confers reliability to scent marks of green lizards and allows their evolution as sexual signals. This result – together with a recent finding about visual female choice in the same species [53] – suggests that female mate choice may be an important agent of sexual selection in this species. Whether high vitamin E levels in nature reflects quality of males or their territories, or acts via conserving other signal molecules in the secretion or by female sensory bias towards vitamin E remains to be tested in the future.
